# Digital Intergenerational Program to Reduce Loneliness and Social Isolation Among Older Adults: Realist Review

**DOI:** 10.2196/39848

**Published:** 2023-01-04

**Authors:** Jie Kie Phang, Yu Heng Kwan, Sungwon Yoon, Hendra Goh, Wan Qi Yee, Chuen Seng Tan, Lian Leng Low

**Affiliations:** 1 Centre for Population Health Research and Implementation SingHealth Regional Health System SingHealth Singapore Singapore; 2 Health Systems and Services Research Duke-NUS Medical School Singapore Singapore; 3 Department of Pharmacy National University of Singapore Singapore Singapore; 4 Internal Medicine Residency SingHealth residency Singapore Singapore; 5 Population Health & Integrated Care Office Singapore General Hospital Singapore Singapore; 6 Saw Swee Hock School of Public Health National University of Singapore Singapore Singapore; 7 Department of Family Medicine & Continuing Care Singapore General Hospital Singapore Singapore; 8 SingHealth Duke-NUS Family Medicine Duke-NUS Medical School Singapore Singapore; 9 Outram Community Hospital SingHealth Community Hospitals Singapore Singapore

**Keywords:** aged, loneliness, older people, review, social isolation

## Abstract

**Background:**

There is a compelling need for an innovative and creative approach to promote social connectedness among older adults to optimize their well-being and quality of life. One possible solution may be through a digital intergenerational program.

**Objective:**

This realist review aimed to identify existing digital intergenerational programs that were used to reduce loneliness or social isolation among older adults and analyze them in terms of strategy, context, mechanisms, and outcomes.

**Methods:**

We performed a realist review with an extensive search of published and gray literature. For scholarly literature, we searched PubMed, Embase, CINAHL, PsycINFO (Ovid), and Social Sciences Citation Index databases for articles published between January 2000 to August 2020. A grey literature search was performed using the Google search engine, and the search was completed in May 2021. We included programs that evaluated digital intergenerational programs for older adults, which described outcomes of loneliness or social isolation. We included quantitative, mixed methods, and qualitative studies, as well as relevant theoretical papers, policy documents, and implementation documents. The studies were appraised based on their relevance and rigor. We synthesized the available evidence from the literature into Strategy-Context-Mechanism-Outcome (S-C-M-O) configurations to better understand what, when, and how programs work.

**Results:**

A total of 31 documents reporting 27 digital intergenerational programs were reviewed. Our final results identified 4 S-C-M-O configurations. For S-C-M-O configuration 1, we found that for community-dwelling older adults, provision of access to and training in digital technology may increase older adults’ self-efficacy in digital devices and therefore increase the use of digital communication with family. In S-C-M-O configuration 2, digital psychosocial support and educational interventions from nurses were found to be useful in reducing loneliness among community-dwelling older adults. In S-C-M-O configuration 3, a video call with a student or family was found to reduce loneliness among older adults residing in long-term residential care facilities. Finally, for S-C-M-O configuration 4, we found that behavioral activation provided through videoconferencing by a lay coach may be useful in reducing loneliness among older adults who are lonely. However, as almost half (11/27, 41%) of the included programs only reported quantitative results, this review focused on screening the discussion section of publications to identify author opinions or any qualitative information to elucidate the mechanisms of how programs work.

**Conclusions:**

This review identified the key strategy, context, and mechanism influencing the success of programs that promote intergenerational interaction through digital means. This review revealed that different strategies should be adopted for different groups of older adults (eg, older adults who are lonely, older adults who reside in long-term residential care facilities, and community-dwelling older adults). The S-C-M-O configurations should be considered when designing and implementing digital intergenerational programs for older adults.

## Introduction

### Background

Driven by decreased fertility rates and increased life expectancy, worldwide population aging is expected to continue [1]. The number of people aged 65 years or older is projected to grow from an estimated 524 million in 2010 to nearly 1.5 billion in 2050, representing around 16% of the total world population in 2050 [2]. Loneliness and social isolation in older adults affect a significant proportion of older adults worldwide, with current estimates of the extent of loneliness among older adults living in the community to be around 50% [3], and around half of people aged >60 years are at risk of social isolation [4]. Loneliness and social isolation pose serious public health risks as they are associated with adverse health outcomes [5,6]. Loneliness may be associated with higher blood pressure, worse sleep, immune stress responses, and worse cognition over time in older adults [7]. A meta-analysis demonstrated that social isolation among older adults significantly increases the likelihood of mortality, and its influence on mortality risk is comparable with well-established risk factors such as smoking, obesity, and physical inactivity [8].

One possible solution to mitigate loneliness and social isolation among older adults may be through an intergenerational program that leverages digital technology [9]. An intergenerational program can be defined as “vehicles for the purposeful and ongoing exchange of resources and learning among older and younger generations for individual and social benefits” [10]. Intergenerational programs can strengthen connections among different age groups and promote organized shared experiences, which may enhance the health of older adults by decreasing the risk of loneliness and social isolation [11-13]. An added advantage of intergenerational interaction over peer interaction is that it provides younger generations with an opportunity to break down agist stereotypes [14], which will help in strengthening community cohesion [15]. Previous reviews have demonstrated the advantage of intergenerational interaction over peer interaction, including allowing younger counterparts to develop new communication skills and improved perceptions toward older adults [9,13,16]. In fact, the Decade of Health Ageing by the World Health Organization has emphasized the need for intergenerational solidarity [17]. Intergenerational programs are usually conducted face to face, and some examples include conducting home visits or organizing large-scale events involving people from different age groups [13,18]. Although there are systematic reviews available that summarize the interventions for reducing social isolation and loneliness in older persons [19-22], these reviews did not focus on intergenerational programs.

During the COVID-19 pandemic, social isolation among older adults intensified with the implementation of social distancing measures [23-26]. Therefore, using digital technology such as video calls to achieve intergenerational bonding becomes more compelling considering the social distancing measures implemented worldwide [27]. The other advantages of digital intergenerational programs in combating loneliness and social isolation among older adults are their ability to connect and reconnect people across large geographic distances, and their support for both synchronous and asynchronous forms of communication [28]. Current reviews of intergenerational programs are primarily based on face-to-face interventions [29-31], with a lack of emphasis on digital interventions. Although there is a scoping review by Reis et al [32] on technologies that foster intergenerational connectivity and relationships, it did not provide an analysis of program outcomes.

In addition, previous traditional reviews tend to predominantly focus on whether the intervention “worked,” often without an understanding of the complexity of the intervention in terms of for whom they may or may not work, under what context and mechanism [33]. The realist review methodology used in this study seeks to provide an explanatory analysis aimed at discerning what works for whom, in what circumstances, in what respect, and how [34]. The emphasis on strategies, contexts, and mechanisms in our realist review can provide an in-depth understanding of how and why interventions are successful or unsuccessful [22,35], which is lacking in existing reviews on intergenerational programs [29,31]. In addition, as studies on intergenerational programs are unlikely to be randomized controlled trials, a realistic review looking at strategy, context, mechanisms, and outcomes will be more appropriate.

### Objective

This review aimed to identify existing digital intergenerational programs used to reduce loneliness or social isolation among older adults and analyze them in terms of strategy, context, mechanisms, and outcomes. The findings of this study will inform the design and implementation of digital intergenerational programs to reduce loneliness or social isolation among older adults.

## Methods

### Overview

**Figure 1:**
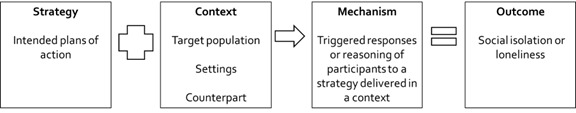
Strategy-Context-Mechanism-Outcome (S-C-M-O) formula.

Our review followed the realist synthesis principles recommended by Pawson and Tilley [36] and was anchored based on the Realist And Meta-narrative Evidence Synthesis: Evolving Standards criteria [37]. In this review, we used the Strategy-Context-Mechanism-Outcome (S-C-M-O) configuration (Figure 1) as this review aimed to understand which digital intergenerational program strategies have been implemented and why some of these strategies were successful [38]. Therefore, the strategies were explicitly identified, along with the context in which they were implemented, the mechanism that was triggered, and which outcome was consequently generated [39]. In this review, we focus on the target population, settings, and counterparts under the context. This S-C-M-O configuration has also been adopted in other realist reviews [40,41].

### Evidence Search

We performed a systematic review of the scholarly and gray literature. We searched the PubMed, Embase, CINAHL, PsycINFO (Ovid), and Social Sciences Citation Index databases for articles published between January 2000 to August 2020. Although intergenerational programs have existed for many decades, most empirical studies assessing the influence of intergenerational interactions on health-related outcomes in older adults have been conducted since 2000 [30,42]. The search in the electronic databases was performed on September 17, 2020. A search strategy with 3 components (ie, “elderly,” “digital communication,” and “intergenerational relationships”), which was devised in collaboration with an information specialist librarian, was utilized (Multimedia Appendix 1). The search filter of the English language was applied when available to minimize potential information loss during the translation process. We downloaded the search records into Endnote and duplicates were removed. A gray literature search was performed using the Google search engine with “intergenerational and elderly and digital” search strings. All 197 results from the Google search engine were screened, and the search was completed on May 2, 2021. Snowball searching was used to identify additional articles based on the reference lists of the included studies and relevant systematic reviews.

Two members of the study team (JKP and HG) independently screened all identified articles. For the scholarly literature, a 2-stage screening process was used where title and abstract were reviewed in the first stage, followed by a review of full-text articles in the second stage. The disagreement rates between the 2 reviewers were 0.14% (6/4382) and 0.9% (2/226) at the title or abstract and full-text screening stages, respectively. For gray literature, the entire document was reviewed because of a lack of executive summary or equivalent in some documents. Any discrepancies in article eligibility were discussed with a third reviewer (YHK) until consensus was reached.

Quantitative, qualitative, and mixed method studies were included. Studies met the inclusion criteria if they described 2-way digital interaction involving older adults with nonfamilial younger generations or with family, were written in English, were evaluative, and described outcomes of interests including loneliness, social isolation, or other related concepts such as social participation and social connectedness. These outcomes are selected given the lack of consistent definition of social isolation in the literature [43] as well as the interchangeable use of “loneliness” and “social isolation” in literature [43]. As there are various definitions of the age range of “older” populations [32,44], a cutoff for the lower age limit was also not specified. As such, we included programs that identified themselves as focusing on older adults or grandparents [32]. The nonfamilial younger generation was defined as either being 30 years old or younger for nonfamily members based on criteria from a previous review [31]. In cases where the characteristics of the intergenerational counterparts were unclear (eg, age of the nurses or coaches involved in communicating with older adults were not clearly described), we contacted the corresponding author to clarify, and only included programs where the counterparts (eg, nurses and coaches) were aged 30 years old or younger. We excluded programs (n=3) [45-47] where the corresponding author did not respond. However, because of the small number of programs focusing solely on digital intergenerational communication (n=1) after an initial review of the literature, we included programs that allow both intergenerational and nonintergenerational digital communication (eg, peer communication). For the familial intergenerational program, we included programs that described digital interaction with family in general, as most of the quantitative studies did not specify the types of digital familial interaction, and this allows a more comprehensive view of the programs available for digital intergenerational communication. More importantly, studies have demonstrated that similar programs are likely to increase contact with younger generations such as children and grandchildren who are well versed with digital technology [48,49]. Descriptive, nonevaluative articles were also included if they were related to a program that had been formally evaluated and included in the review. Articles that were not program specific (eg, commentaries or discussion papers) were excluded.

### Data Extraction and Appraisal of Studies

Relevant information from the documents was extracted using a data extraction template. The studies were appraised based on their relevance and rigor. Relevance was defined as the level of contribution to the review, and rigor was defined by the methodological quality of a study conducted on a digital intergenerational program. Relevance was assessed by reviewing the details provided for (1) context (eg, user, program features, or design components), (2) mechanism: hypotheses as to how specific strategy worked or did not work, and (3) outcome: reasons for effect or lack of effect on outcomes related to loneliness or social isolation. These details were obtained by reviewing the documentation of usability evaluation, program or study protocols, and publications related to evaluations (eg, clinical intervention studies evaluating efficacy or effectiveness). In programs where authors did not describe how they thought their program worked or did not work, this was inferred by the study team after careful reading of the description of the program. The relevance was rated as low (little or no information), medium (some information), and high (well-described information). The criteria for assessment of relevance were adopted from a previous realist review [33], in which “strategy” was considered in the mechanism section. The methodological quality of evidence (rigor) around each therapy was assessed using the Mixed Methods Appraisal Tool (MMAT) [50]. The MMAT assesses the quality of qualitative, quantitative, and mixed methods studies. It focuses on methodological criteria and includes five core quality criteria for each of the following five categories of study design: (1) qualitative, (2) randomized controlled, (3) nonrandomized, (4) quantitative descriptive, and (5) mixed methods.

### Evidence Synthesis

We examined the strategy, context, mechanism, and outcome in each program and looked for recurrent patterns of outcomes and their associated strategies, contexts, and mechanisms. We concentrated on what appeared to be recurrent patterns of contexts and outcomes in the data and then sought to explain them through the strategies and mechanisms by which they occurred. The proposed S-C-M-O configurations were analyzed at different levels of abstraction (within and across programs) to determine the most robust and plausible explanations of how, in a context, with the strategy and mechanism, the outcomes observed could be generated. The evaluation of relevance and rigor was considered when generating and revising S-C-M-O configurations. The initial list of S-C-M-O configurations was revised based on the consensus between study team members, based on the synthesis process recommended by Pawson [51], including synthesis to adjudicate between rival program theories and synthesis to consider the same theory in comparative settings.

## Results

### Overview

**Figure 2:**
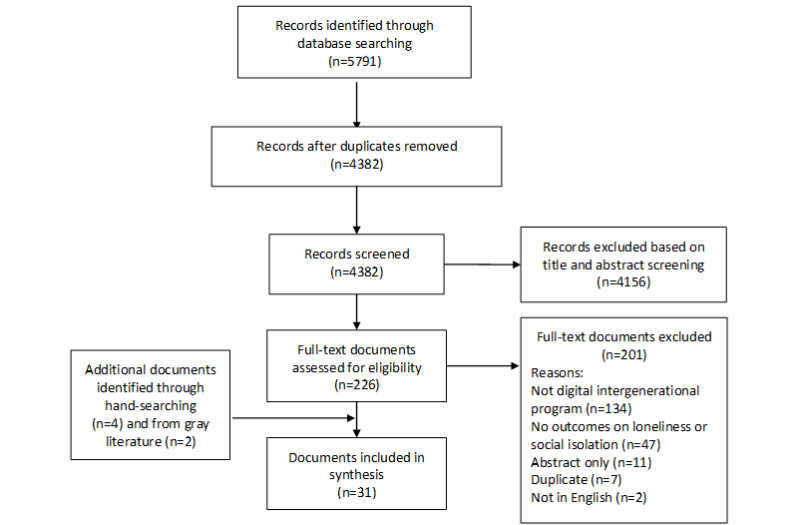
Preferred Reporting Items for Systematic Reviews and Meta-Analyses (PRISMA) flow diagram.

Figure 2 presents a flow diagram outlining the evidence-based search process. We retrieved 5791 records from the scholarly literature search of 5 databases (PubMed, CINAHL, PsycINFO, and Social Sciences Citation Index databases). After removing duplicates, a total of 4382 unique and potentially eligible documents were reviewed for inclusion. We excluded 4156 records and 201 documents at the title or abstract and full-text screening, respectively. The reasons for exclusion at the full-text screening stage can be found in Multimedia Appendix 2. We added 4 documents from the snowball searching method based on the reference lists of already included studies and relevant systematic reviews. In addition, we also retrieved 2 relevant documents from the grey literature search using the Google search engine. In total, 31 documents from the scholarly and grey literature search detailing 27 unique digital intergenerational programs were included for synthesis in this realist review.

### Structure and Delivery Features

Table 1 presents an overview of the structure and delivery features of the 27 programs. A total of 10 programs were for older adults residing in long-term residential care [52] (including nursing home [53-55], retirement homes [56], aged care facilities [57], assisted living retirement facilities [58], social housing [59], care homes [60], and veterans' care facility [61]), 16 programs for community-dwelling older adults [62-75], and 2 programs included both community-dwelling older adults and older adults residing in long-term residential care facilities [48,76]. Only 1 program (StoryBox) was designed for exclusive digital intergenerational interactions [70]. Most (8/27, 30%) programs were conducted in the United States [53,58,60,66,72,74,76,77].

**Table 1:** Structure and delivery characteristics of digital intergenerational program.

Program, country	Participants					Program detail				
	Age (years)	Older adults, n	Lonely or socially isolated	Settings	Duration		Strategy	Intergenerational component	Device	Training for older adult
ACTION [62], Norway	57-85	19	Not specified	Community- dwelling	12 months		Participants received a modern broadband-linked PC, and an ICT^a^ course consisting of three 3-hour classes dispersed over a 3-week period.	Email with grandchildren	Computer	Yes
ACTION (redesigned) [63], Sweden	66-85	8	Not specified	Community- dwelling	Not specified		The app integrated a web-based multimedia system and the video communication system into a single user interface. Users could access a variety of multimedia information programs in the ACTION database and use the videoconferencing device for consultation and social purposes.	Video call with family	Computer	Yes
ACTIVE [52], Norway	Mean 78.3 (SD 12.5)	15	Not specified	Long-term residential care	1 year		Participants were provided with an internet connected tablet, free of charge, to use as they liked for an unlimited period. The iPad was set up with an individual user account, including email, Apple-ID, Skype-ID, passwords, and codes.	Messaging, video call, and email with younger generations of family, for example, grandchildren	Apple iPad	Yes
AGES 2.0 [48], United Kingdom	60-95	53	Not specified	Community- dwelling and long-term residential care (care homes)	12 months		Participants received a customized computer platform with a simplified touchscreen interface (“EasyPC”) and any necessary broadband infrastructure. “Care technologists” administered the training.	Digital interaction (email, Skype, or Facebook) with younger generations of family, for example, children and grandchildren	Customized computer platform with a simplified touchscreen interface	Yes
AO [73], Australia	58-81	7	Yes	Community- dwelling	8 months		Participants were provided Apple iPad with cellular access, along with vouchers for data access throughout the project, and App Store card for buying apps.	Messaging with younger generations of family, for example, children	Apple iPad	Yes
Collage and storytelling [71], Australia	Not specified	3	Not specified	Community- dwelling	3 weeks		The system used combines the “Collage” component and the “Storytelling” component.	Digital intergenerational play and storytelling with grandchildren	Touch screen monitor	No
Demiris et al [58], United States	>65	4	Not specified	Long-term residential care (assisted living retirement facility)	3 months		The videophone can display 3 kinds of real-time images during a video call: self, other party, and a combination of both, depending on user preference. It plugs into a regular telephone and does not interfere with its use. A video call is possible only when both parties have videophone units and consent to a video call.	Video call and email with younger generations of family, for example, grandchildren	Video-telephone	No
Digital age [59], Northern Ireland	Not specified	82	Not specified	Long-term residential care (social housing)	10 weeks		Digital Age consisted of a free, in-house, 10-week IT course for residents. The program also provided free IT hardware for each participating housing scheme, free web-based digital toolkits for older learners and their supporters, and a series of intergenerational digital projects to encourage links between older and younger people, further develop residents’ digital capabilities and help to sustain the program beyond the program lifetime.	Video call with family	Not specified	Yes
Esc@pe [75], Netherlands	Mean 66	12	Yes	Community- dwelling	3 years		At the start of the project, participants were given five 2-hour lessons at home by experienced teachers. During these lessons, the participants learned how to email and how to use the internet. During the rest of the project, the participants were supported and coached by visiting volunteers who had also paid home visits to the participants once every 2 or 3 weeks before the start of the pilot project.	Using internet and email to communicate with family	Computer	Yes
InTouch [61], Canada	Mean 92.2 (SD 3.0)	11	Not specified	Long-term residential care	12 weeks		Veteran and volunteer participants were each given an iPad with the InTouch app on it, as well as a detailed instructions manual.	Using InTouch app to communicate with family	Apple iPad	Yes
LINE [54], Taiwan	Mean 81.1 (SD 8.5)	32	Not specified	Long-term residential care (nursing home)	6 months		Participants interacted with their family members once a week for 6 months using a smartphone and the “LINE” app. Discussion topics were provided to nurses and the participants, such as their meals, organized activities, and “news” on nursing home life.	Video call with family	Smartphone	No
Loi et al [57], Australia	Mean 69.9	5	Not specified	Long-term residential care (aged care facility)	6 weeks		Structured 6-week, twice weekly program of 45-minute duration based on a local program was used for older adults (internet for Seniors). Apple iPads were used.	Digital interaction with family	Apple iPad	Yes
Media Parcels [64], United Kingdom	82	1	Not specified	Community- dwelling	Not specified		A facilitator, upon specific requests to participants, collects media and wraps them in text commentary, bringing out their memories and meaning. Next, the facilitator passes the wrapped media parcel to a target person, who in turn unwraps them.	Digital interaction with children	Not specified	Not specified
MSN^b^ or Skype [55],Taiwan	Mean 74.4 (SD 10.2)	24	Not specified	Long-term residential care (nursing home)	3 months		The videoconference program was designed for once a week (the in-person visiting frequency for most families) and to last for 3 months to provide time for adjustment to a new program. The residents were helped to use the videoconference technology by a trained research assistant, who spent at least 5 minutes per week with the residents at the appointment time.	Video call with family	Computer	No
Neves et al [56], Canada	74-95	12	Not specified	Long-term residential care (retirement home)	3 months		The app allowed residents to send and receive photos, audio, video, and text messages with sent messages being predefined to increase simplicity. The residents’ contacts could respond using their own emails and devices.	Using app to communicate with younger generations of family, for example, children	Apple iPad	Yes
Plymouth SeniorNet [65], United Kingdom	One-to-one help: mean 79.0 (SD 7.5); group help: mean 74.3 (SD 8.2)	144	Not specified	Community- dwelling	Depends		Sessions by volunteers covered basic computer use, how to get on the web and search the internet, shopping, email, Skype or FaceTime, and web-based news and entertainment.	Digital interaction with family	Computer	Yes
PRISM [66], United States	Mean 76.9 (SD 7.3)	300	Not specified	Community- dwelling	12 months		PRISM software app included internet access, an annotated resource guide, a dynamic classroom feature, a calendar, a photo feature, email, games, and web-based help.	Digital interaction with family	Computer	Yes
Skype [53], United States	71-97	40	Not specified	Long-term residential care (nursing home)	14 weeks		The Skype videoconferencing intervention took place on a weekly for a total of 10 sessions over a 14-week period in a private room at the nursing home.	Video call with family	Computer	No
Skype on Wheel [60], United States	Not specified	20	Not specified	Long-term residential care (care home)	6 weeks		Students from local school and older adults across 3 care homes in engaged in Skype video calls over a 6-week study. Residents were supported by care staff; students accessed Skype from school laptops. A conversational aid was trialed with students to assist their conversation with an older generation.	Video call with students	Wheeled device that could hold an iPad and handset	No
StoryBox [70], country not specified	63-76	8	Not specified	Community- dwelling	2-4 weeks		StoryBox alleviates the barriers of communication between different generations. For young grandchildren, this often means the sharing of crafts, drawings, stickers, and short exclamations. For grandparents, the device provides a way to digitize analog memories and use handwriting for communication.	Digital sharing of photos and audio recordings with grandchildren	Smartphone and tablet	No
Tech Allies [77], United States	Mean 75 (SD 7.9)	83	Yes	Community- dwelling	2 months		Participants took part in 8 weekly, 1:1 digital training sessions. Participants each received a tablet, a tablet case, a stylus, broadband access or a hot spot device, and a certificate of completion at the end of the program.	Digital interaction with family	Tablet	Yes
Tele-BA [74], United States	Mean 74.4 (SD 8.2)	43	Yes	Community- dwelling	12 weeks		Lay counselors delivered videoconference behavioral activation	Videoconference behavioral activation by lay counselors	Computer	No
Telesenior [67], Belgium	Mean 72 (SD 9.3)	71	Not specified	Community- dwelling	Not specified		The telenurses delivered psychosocial support and educational interventions based on 3 principles: contact and communication, safety and protection, and care mediation.	Video call with nurse	Video-telephone	No
Tlatoque [68], Mexico	Not specified	2	Not specified	Community- dwelling	21 weeks		Tlatoque communicates to Facebook site to expose photographs in the participant’s home and provides means of reciprocating information into Facebook.	Digital interaction with younger generations of family, for example, children and grandchildren	Digital picture frame with wireless capabilities or PC with multitouch screen	Yes
White et al [76], United States	Mean 71 (SD 12)	48	Not specified	Community- dwelling and long-term residential care (nursing facility)	5 months		Participants received 9 hours of small group training in 6 sessions over 2 weeks. Computers were available for continued use over 5 months and the trainer was available 2 hours per week for questions.	Digital interaction with family	Computer	Yes
Williams et al [72], United States	Phase 1: 59-86; phase 2 54-82	Phase 1: 16; phase 2: 29	Yes	Community- dwelling	2 weeks		The course on computer-mediated communication lasted for six 2- hour classes. Each course followed the same module: introduction to computer-mediated communication, email and instant messaging, microblogging: Twitter, social networking sites: Facebook, video chat, and web-based safety.	Digital interaction with family	Computer	Yes
You, me and television [69], Portugal	65-73	3	Not specified	Community- dwelling	3-6 weeks		The system has three main features: (1) user feed; (2) managing groups of friends; and (3) photo viewing and sharing.	Digital interaction with family	Television	Yes

^a^ICT: information and communications technology.

^b^MSN: Microsoft Network.

### Level of Contribution and Methodological Quality

An overview of the level of contribution and methodological quality of the documents elaborating the 27 programs is presented in Table 2. On the basis of the level of contribution assessment, 20 programs were rated as having a high level of contribution to the context, mechanism, and outcome. Across the programs, descriptions of mechanisms and outcomes were less developed than descriptions of contexts. All studies met at least 2 of the 5 MMAT criteria. The detailed MMAT appraisal of documents evaluating the digital intergenerational program is shown in Multimedia Appendix 3.

**Table 2:** Level of contribution and methodological quality.

Program	Quality appraisal				Associated MMAT^a^ scores
	Context	Mechanism^b^	Outcome		
ACTION	High	Low	Low	5 [62]	
ACTION (redesigned)	High	Low	Low	2 [63]	
ACTIVE	High	Medium	Low	5 [52]	
AGES 2.0	High	High	High	3 [48]	
AO	High	High	Medium	5 [73]	
Collage and storytelling	High	High	High	4 [71]	
Demiris et al	High	Medium	Low	4 [58]	
Digital age	High	High	Low	5 [59], 5 [78]	
Esc@pe	High	High	High	5 [75]	
InTouch	High	High	High	5 [61]	
LINE	High	High	High	5 [54]	
Loi et al	High	High	Low	4 [57]	
Media parcels	High	High	High	5 [64]	
MSN^c^ or Skype	High	High	High	5 [55], 5 [79]	
Neves et al	High	High	High	5 [56]	
Skype	High	High	High	5 [53]	
Skype on Wheel	High	High	High	5 [60], 5 [49]	
StoryBox	High	High	High	2 [70]	
Tech Allies	High	High	High	3 [77]	
Tele-BA	High	High	High	3 [74], 3 [80]	
Telesenior	High	High	High	4 [67]	
Tlatoque	High	High	High	5 [68]	
PRISM	High	High	High	4 [66]	
White et al	High	High	High	3 [76]	
Williams et al	High	High	High	5 [72]	
Plymouth SeniorNet	High	High	High	5 [65]	
You, me and television	High	High	High	2 [69]	

^a^MMAT: Mixed Methods Appraisal Tool.

^b^Consists of strategy and mechanism.

^c^MSN: Microsoft Network.

### S-C-M-O Configurations

**Figure 3:**
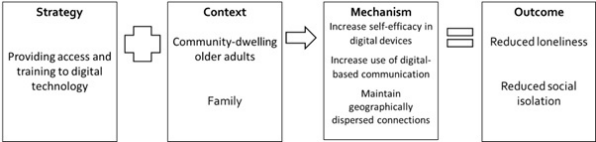
Strategy-Context-Mechanism-Outcome configuration 1 involving provision of access and training to digital technology for community-dwelling older adults.

**Figure 4:**
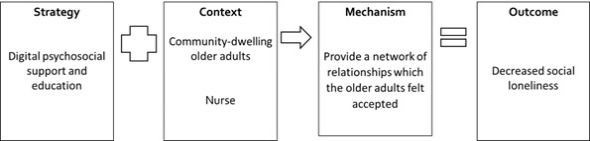
Strategy-Context-Mechanism-Outcome configuration 2 involving provision of digital psychosocial support and education by nurses for community-dwelling older adults.

**Figure 5:**
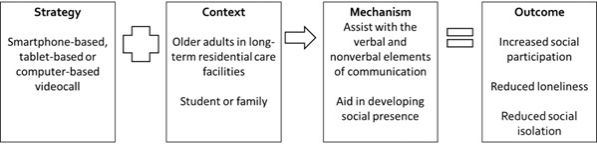
Strategy-Context-Mechanism-Outcome configuration 3 involving video call with older adults in long-term residential care facilities.

**Figure 6:**
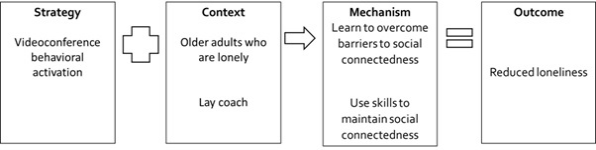
Strategy-Context-Mechanism-Outcome configuration 4 involving videoconference behavioral activation for older adults who are lonely.

Of the candidate S-C-M-O configurations based on the authors’ description (Multimedia Appendix 4), 4 S-C-M-O configurations were substantively supported by the available evidence (Figures 3-6). We present the configurations with key examples of strategies, contexts, mechanisms, and outcomes from the reviewed documents. S-C-M-O configurations 1 and 2 focused on community-dwelling older adults, S-C-M-O configuration 3 focused on older adults residing in long-term residential care facilities, and S-C-M-O configuration 4 focused on older adults who are lonely.

### S-C-M-O Configuration 1

A total of 4 programs contributed to this S-C-M-O configuration: ACTION [62], Plymouth SeniorNet [65], PRISM [66], and Tlatoque [68]. For community-dwelling older adults, provision of access to and training in digital technology may increase their self-efficacy in digital devices, thereby increasing the use of digital-based communication with family (Figure 3). The outcomes observed for the 4 programs included reduced loneliness [65,66], reduced social isolation [66], and increased frequency of contact [62,68].

In all 4 programs, the devices were provided free of charge for older adults. Of the 4 programs, 2 (PRISM [66] and Tlatoque [68]) used apps or systems specially designed for older adults, which may have “eased the adoption of the technology” [68]. The other programs used commercially available digital communication modes, for example, email (n=2) [62,65] and Skype (n=1) [65].

The mode of training included one-to-one, group, and a combination of one-to-one and group training. In the Plymouth SeniorNet program, older adults attending group sessions appeared to have a greater reduction in loneliness as compared with those in one-to-one sessions, although the results from the two modes of training may not be comparable, as the allocation was not random [65]. Participants in the Plymouth SeniorNet program also mentioned that training conducted by someone closer to their age was important [65].

### S-C-M-O Configuration 2

One program (Telesenior [67]) contributed to the S-C-M-O configuration. For community-dwelling older adults, digital psychosocial support and educational interventions from nurses were useful in reducing loneliness (Figure 4). In the Telesenior program, digital psychosocial support and educational interventions were delivered through video-telephone to homebound older adults based on 3 principles: contact and communication, safety and protection, and care mediation [67]. The digital psychosocial support and educational interventions from nurses can provide “a network of relationships which the older adults felt accepted, had common interests and concerns, and found help, advice, and support” [67]. In the Telesenior program, older adults who were older (>66 years old), were widowed, lived alone, had financial problems, and used several health and social services showed improvement in feelings of social loneliness after participating in the program [67].

### S-C-M-O Configuration 3

A total of 7 programs—ACTIVE [52], Demiris et al [58], Digital Age [59], LINE [54], Microsoft Network (MSN) or Skype [55], Skype [53], and Skype on Wheel [60]—contributed to this S-C-M-O configuration. In this review, we found that video calls with students or families may be useful in reducing loneliness among older adults residing in long-term residential care facilities (Figure 5). Only 1 program (Skype on Wheel [60]) evaluated intergenerational communication with students from a local school, whereas the other 6 programs (LINE [54], MSN or Skype [55], Skype [53], ACTIVE [52], Demiris et al [58], and Digital Age [59]) were designed to facilitate communication with family members or friends of older adults in long-term residential care facilities. It has been hypothesized that a video call helps in language interaction as well as verbal and nonverbal elements of communication. Video calls may also aid in promoting a social presence for older adults and family members [58]. The outcomes observed for the 7 programs included reduced loneliness [53,54,58,79], reduced social isolation [58], and improved social participation [52,59,60].

A total of 4 programs used existing software programs, including LINE [54], MSN [55], and Skype [52,53,55] for video calls, whereas 1 program used videophones [58]. For programs using commercially available software, smartphone [54], tablet [52,60] and laptop [55] have been used. The frequency of contact between older adults and their families was designed to be once per week in 4 programs—LINE [54], MSN or Skype [55], Skype [53], and Demiris et al [58].

As highlighted in the Skype on Wheel [60] program, “younger generations (grandchildren) may not be sure of how to communicate with their elderly relatives”; therefore, it may be helpful to provide conversational aid to facilitate intergenerational communication, such as a list of possible conversational topics as seen in 2 programs (Skype on Wheel [60] and LINE [54]). Although not developed for older adults residing in long-term residential care facilities, other programs have investigated digital storytelling [70] and exposure to photographs in the older adults’ environment [68] as ways to facilitate intergenerational conversation.

A total of 2 programs (ACTIVE [52] and Digital Age [59]) explicitly included training on using digital technology for older adults residing in long-term residential care facilities. In the ACTIVE program, the authors highlighted that “a carefully selected, smaller set of basic apps was installed when the intervention started” to avoid overwhelming the older adults [52]. The content of the training is well described in the Digital Age program [59], which includes the following core subjects: learning how to use a tablet, browsing the internet, staying safe on the internet, emailing, using an App Store, and video calling. The content of the training sessions was flexible and tailored to the needs of older adults in the Digital Age program [59].

### S-C-M-O Configuration 4

One program, Tele-Behavioral Activation (BA) [74], contributed to this S-C-M-O configuration. We found that behavioral activation provided through videoconferencing by a lay coach may be useful in reducing loneliness among older adults who are lonely (Figure 6). Several studies have evaluated the effectiveness of digital training courses [72,73,75,77] in reducing loneliness or social isolation for older adults who were lonely or socially isolated. However, as highlighted in the Assertive Outreach (AO) program, establishing “even a small web-based social network proved very difficult in many cases” for older adults who were socially isolated, which may have resulted in the lack of improvement in the outcomes in most of these studies [73]. BA is a brief, structured behavioral approach that aims to increase and reinforce wellness-promoting behaviors that can be conducted by lay coaches [74]. In the Tele-BA program, lay coach “worked with participants to identify and schedule value-based activities, rewarding social engagement and activities, and using strategies to reduce and solve barriers to social connectedness [74]. Participants first reviewed their daily activity patterns, then chose activity goals, worked on specific implementation plans, and reviewed their successes and areas for improvement” [74]. This may have enabled older adults to learn to overcome barriers to social connectedness and to use skills for maintaining social connectedness over time, leading to reduced levels of loneliness that were sustained beyond the 5 sessions of tele-BA.

## Discussion

### Principal Findings

In this review, we sought to answer the following question: “How do different digital intergenerational programs interact with different contexts to produce certain outcomes?” This review revealed that different strategies should be adopted for different groups of older adults (eg, older adults who are lonely, older adults who reside in long-term residential care facilities, and community-dwelling older adults). For example, providing training and access to digital technology may be useful in reducing loneliness among community-dwelling older adults but not for older adults who are already lonely or socially isolated. This may be because establishing “even a small web-based social network proved very difficult in many cases” for older adults who are socially isolated as discussed in the AO program [73]. Similar to AO, Tech Allies program also pointed out the older adults “were already facing many contextual factors in their daily lives, such as physical disability and a lack of close friends and living relatives” would make “their loneliness more systemic and harder to change” [77]. Although tele-BA by lay coaches may be helpful for lonely older adults, future studies should explore different program strategies for this subgroup of older adults with more complex needs. A possibility is to entail young volunteers to befriend older adults who lack existing social support [81]. Williams [72] investigated the effect of a 2-week computer-mediated communication course for lonely older adults and found “no significant difference in loneliness between pre-test and post-test” [72]. However, as explained by the author, the lack of observed differences after the intervention was not unexpected with the short duration of the intervention (2 weeks) [72]. Therefore, the duration of intergenerational programs should be considered before implementation.

Among the digital intergenerational programs included in this realist review, 2 programs (AGES 2.0 [48] and White et al [76]) targeted both community-dwelling older adults and older adults residing in long-term care facilities by providing training in digital technology. However, both the programs demonstrated unsuccessful outcomes [48,76]. A possible reason for the unsuccessful outcome from these 2 programs may be that although providing training in digital technology may be useful to reduce loneliness among community-dwelling older adults based on S-C-M-O configuration 1 (all 4 programs in S-C-M-O configuration 1 achieved successful outcomes), this program strategy may not be useful for older adults in long-term residential care facilities. This further supports the importance of designing targeted digital intergenerational programs for different groups of older people (eg, older adults residing in long-term residential care facilities and community-dwelling older adults). The AGES 2.0 study also found that “feelings of self-competence, social engagement, and maintenance of identity were critical to the intervention’s success” [48]. Future research should explore whether interventions that enhance these aspects are useful in promoting social connectedness among older adults.

### Comparison With Prior Work

In a previous realistic review exploring the use of technology to engage hospitalized patients, the authors found that a user-centered design may increase the engagement level [82]. However, in our realistic review of digital intergenerational programs based on digital technology, only 3 programs designed for community-dwelling older adults incorporated a user-centered design [63,66,69], and 2 programs were evaluated in a small sample (n<10) [63,69]. Studies in long-term residential care facilities using existing digital communication tools such as LINE and Skype demonstrated beneficial effects on reducing loneliness [54,55], which implies that user-centered design may not be critical for the success of digital intergenerational design. However, this could be because staff are available at long-term residential care facilities to assist with the set-up of the video call tools in these programs, which facilitates intergenerational communication with family members or students [54,55,60] and mitigates the potential problem of digital illiteracy among older adults [83,84]. Future research should be conducted to examine whether a user-centered design may have contributed to a reduction in loneliness or social isolation among specific groups of older adults.

### Strengths and Limitations

This review is the first to use a realist framework to study digital intergenerational programs for older adults. The realist framework allowed us to consider empirical findings and theories together to understand how these programs worked. Previous reviews on the effectiveness of intergenerational programs have focused primarily on scholarly literature [29-31] and, therefore, have provided limited insight into the complex causal pathways that may underpin the efficacy or effectiveness of intergenerational programs. The inclusion of diverse research designs, such as quantitative, qualitative, and mixed methods studies, enabled this review to leverage the strengths of each approach. From a realistic perspective, this diversity has huge explanatory value and can help uncover contexts and mechanisms not typically captured in traditional systematic reviews and meta-analyses [33].

However, a limitation of this realist review is that nearly half (11/27, 41%) of the programs reported only quantitative results. A problem with conducting a realist review of quantitative studies is that their primary emphasis is on quantitative results; thus, there may be fewer descriptions and explanations of the mechanisms [85]. Thus, our realist review generally focuses on screening the discussion section of publications to identify author opinions or any qualitative information that may provide information on the mechanisms of how certain programs work. As we inferred most of the information regarding the mechanism from the authors’ comments and discussions in the quantitative studies, we acknowledge the subjectivity of these inferences. Nevertheless, the S-C-M-O configurations derived from this study may serve as a basis for further studies to corroborate the proposed theory and mechanisms that drive program outcomes in different contexts.

Second, we acknowledge that for some programs, the outcome observed may not be solely attributable to intergenerational interaction, as the participants may interact with their peers or spouses using digital technology. We decided to include these studies, as there was only 1 study [70] focusing solely on intergenerational interaction using digital technology. However, the inclusion of programs that accommodate both nonintergenerational and intergenerational communication provides a more comprehensive list of programs available for intergenerational communication. As such, some programs in this review may need to be adapted for intergenerational interactions only, and their effectiveness in addressing isolation and loneliness may require investigation in future studies.

Third, as the search in the scholarly literature was restricted to articles published before August 2020, our review may have excluded studies published after the cutoff date. Nevertheless, the findings of this review can serve as a foundation for future research on digital intergenerational programs.

Finally, another limitation of this study was the inclusion of only English-language documents, which may have potentially led to the omission of relevant programs from English-speaking countries. Among the 27 programs included in this review, only 2 (7%) programs were conducted in Asian countries [54,79]. However, the inclusion of only English-language documents minimizes potential information loss during translation.

### Conclusions

This review identifies the key strategy, context, and mechanism that influence the success of programs in reducing loneliness or isolation among older adults by potentially promoting intergenerational interaction through digital means. Digital interventions are becoming increasingly popular to tackle social problems, such as loneliness and social isolation. We identified 4 S-C-M-O configurations to consider when developing intergenerational programs for older adults. Future studies, especially quantitative studies, should consider clearly describing the components of the program and their corresponding contexts and mechanisms driving the improvement of outcomes in digital intergenerational programs. With a better understanding of the components and mechanisms of digital intergenerational programs, well-informed decisions can be made when planning or developing digital intergenerational programs.

## Appendix

Multimedia Appendix 1 Search strategies in databases.

Multimedia Appendix 2 List of studies excluded during full text screening.

Multimedia Appendix 3 Mixed Methods Appraisal Tool appraisal for documents evaluating digital intergenerational program.

Multimedia Appendix 4 Candidate Strategy-Context-Mechanism-Outcome configurations for digital intergenerational programs based on the authors’ description.

## Abbreviations

AO: Assertive Outreach
BA: Behavioral Activation
MMAT: Mixed Methods Appraisal Tool
MSN: Microsoft Network
S-C-M-O: Strategy-Context-Mechanism-Outcome

## Data Availability

The data sets generated during and/or analyzed during this study are available from the corresponding author upon reasonable request.
