# Supplementary material for: Digital Intergenerational Program to Reduce Loneliness and Social Isolation Among Older Adults: Realist Review
Source: JMIR Aging. 2023 Jan 4;6:e39848. doi: 10.2196/39848 (PMC9850285; doi:10.2196/39848)
Supplement: Multimedia Appendix 1 [file aging_v6i1e39848_app1.docx]

Supplementary table 1: Search strategies in databases

| **Construct** | **PubMed** | **Embase** | **PsycINFO** | **CINAHL** | **SSCI** |
| --- | --- | --- | --- | --- | --- |
| Elderly | (((("elder*"[Title/Abstract] OR "aging"[Title/Abstract] OR "older"[Title/Abstract] OR "retire*"[Title/Abstract] OR "senior*"[Title/Abstract]) OR "nursing home*"[Title/Abstract] OR "retirement home*"[Title/Abstract]) OR (Aged[MeSH Terms])) | (elder*:ab,ti OR ag$ing:ab,ti OR older:ab,ti OR retire*:ab,ti OR senior*or:ab,ti OR 'nursing home*':ab,ti OR 'retirement home*':ab,ti OR 'aged'/exp) | Elder* or Ag?ing or older or retire* or senior* OR Nursing home* or retirement home* OR exp Geriatrics/ | (Elder* or Ag#ing or older or retire* or senior* OR “Nursing home*” or “retirement home*”) OR MH Aged | Elder* OR “Ag$ing” OR older OR retire* OR senior*OR “Nursing home*” OR “retirement home*”) |
| Digital communication | Mobile[Title/Abstract] OR cell*[Title/Abstract] OR digital[Title/Abstract] OR smart[Title/Abstract] OR information[Title/Abstract]) AND (device[Title/Abstract] OR device-based[Title/Abstract] OR phone[Title/Abstract] OR technolog*[Title/Abstract] OR technology-based[Title/Abstract])) OR infotech [Title/Abstract] OR ‘social media’[Title/Abstract] OR ‘electronic communication’[Title/Abstract] OR ‘online interaction’[Title/Abstract] OR ‘social network’[Title/Abstract] OR "voice over internet protocol"[Title/Abstract] OR VoIP[Title/Abstract] OR "video call*"[Title/Abstract] OR videoconferenc*[Title/Abstract] OR video conferenc*[Title/Abstract] OR "videophone communication"[Title/Abstract] OR viber[Title/Abstract] OR "viber app"[Title/Abstract] OR skype[Title/Abstract] OR video call*[Title/Abstract] OR facetime[Title/Abstract] OR WhatsApp[Title/Abstract] OR ICTs[Title/Abstract] OR iPAD[Title/Abstract] OR iPhone[Title/Abstract] OR "google hangouts"[Title/Abstract] OR "virtual communication*"[Title/Abstract] OR "online communication*"[Title/Abstract] OR "tablet device*"[Title/Abstract] OR "tablet comput*"[Title/Abstract]) OR (Cell phone[MeSH Terms])) OR (mobile applications[MeSH Terms])) OR (online social networking[MeSH Terms])) OR (videoconferencing[MeSH Terms])) OR (Computer Communication Networks[MeSH Terms] | ((((mobile OR cell* OR digital OR smart OR information) NEAR/3 (device OR 'device based' OR phone OR technolog* OR 'technology based')):ab,ti) OR infotech:ab,ti OR 'social media':ab,ti OR 'electronic communication':ab,ti OR 'online interaction':ab,ti OR 'social network':ab,ti OR 'voice over internet protocol':ab,ti OR voip:ab,ti OR videoconferenc*:ab,ti OR 'video conferenc*':ab,ti OR 'videophone communication':ab,ti OR viber:ab,ti OR 'viber app':ab,ti OR skype:ab,ti OR 'video call*':ab,ti OR facetime:ab,ti OR whatsapp:ab,ti OR icts:ab,ti OR ipad:ab,ti OR iphone:ab,ti OR 'google hangouts':ab,ti OR 'virtual communication*':ab,ti OR 'online communication*':ab,ti OR 'tablet device*':ab,ti OR 'tablet comput*':ab,ti OR 'mobile phone'/exp OR 'mobile application'/exp OR 'social media'/exp OR 'computer network'/exp OR 'videoconferencing'/exp) | ((Mobile or cell* or digital or smart or information) adj3 (device or device-based or phone or technolog* or technology-based)) or infotech or social media or electronic communication or online interaction or social network OR voice over internet protocol OR VoIP OR video call* OR videoconferenc* OR video conferenc* OR videophone communication OR viber OR viber app OR skype or video call* OR facetime OR WhatsApp OR ICTs OR iPAD OR iPhone OR google hangouts OR virtual communication* OR online communication* OR tablet device* OR tablet comput* OR exp Mobile devices/ OR exp Mobile applications/ OR exp Social media/ OR exp videoconferencing/ OR exp computer mediated communication/ | (Mobile or cell* or digital or smart or information) N3 (device or device-based or phone or technolog* or technology-based)) or infotech or "social media" or "electronic communication" or "online interaction" or "social network“ or “voice over internet protocol” OR VoIP OR “video call*” OR videoconferenc* OR “video conferenc*” OR “videophone communication” OR viber OR “viber app” OR skype or “video call*” OR facetime OR WhatsApp OR ICTs OR iPAD OR iPhone OR “google hangouts” OR “virtual communication*” OR “online communication*” OR “tablet device*” OR “tablet comput*”) OR MH Cellular phone OR MH mobile applications OR MH social media OR MH online social networking OR MH Computer Communication Networks OR MH videoconferencing | Mobile OR cell* OR digital OR smart OR information)   NEAR/3  (device OR device-based OR phone OR technolog* OR technology-based))  OR  infotech  OR  "social  media"  OR  "electronic  communication"  OR  "online  interaction"  OR  "social  network“OR “video call*” OR videoconferenc* OR “video conferenc*” OR “videophone communication” OR viber OR “viber app” OR skype or “video call*” OR facetime OR WhatsApp OR ICTs OR iPAD OR iPhone OR “google hangouts” OR “virtual communication*” OR “online communication*” OR “tablet device*” OR “tablet comput*” |
| Intergenerational | soci*[Title/Abstract]) AND (connect*[Title/Abstract] OR inclu*[Title/Abstract] OR isolate*[Title/Abstract] OR distanc*[Title/Abstract] OR engag*[Title/Abstract])) OR ((intergeneration[Title/Abstract] OR inter-generation*[Title/Abstract] OR cross-generation*[Title/Abstract] OR multigenerational[Title/Abstract] OR multi-generation*[Title/Abstract]) AND (program*[Title/Abstract] OR interaction[Title/Abstract]))) OR loneliness[Title/Abstract] OR (Social isolation[MeSH Terms])) OR (intergenerational relations[MeSH Terms] | soci* NEAR/3 (connect* OR inclu* OR isolate* OR distanc* OR engag*)):ab,ti) OR (((intergeneration OR 'inter generation*' OR 'cross generation*' OR multigenerational OR 'multi generation*') NEAR/3 (program* OR interaction)):ab,ti) OR loneliness:ab,ti OR 'social isolation'/exp) | ((Soci* adj3 (connect* or inclu* or isolate* or distanc* or engag*)) or ((intergeneration or inter-generation* or cross-generation* or multigenerational or multi-generation*) adj3 (program* or interaction)) OR loneliness)  Or exp Social isolation/ OR exp intergenerational relations/ | ((Soci* N3 (connect* or inclu* or isolate* or distanc* or engag*)) or ((intergeneration or inter-generation* or cross-generation* or multigenerational or multi-generation*) N3 (program* or interaction)) or loneliness) OR MH  Social isolation OR MH intergenerational relations | Soci* NEAR/3 (connect* OR inclu* OR isolate* OR distanc* OR engag*))  OR  ((intergeneration OR inter-generation* OR cross-generation* OR multigenerational OR multi-generation*)   NEAR/3  (program* OR interaction)) OR loneliness) |
