# Supplementary material for: Digital Intergenerational Program to Reduce Loneliness and Social Isolation Among Older Adults: Realist Review
Source: JMIR Aging. 2023 Jan 4;6:e39848. doi: 10.2196/39848 (PMC9850285; doi:10.2196/39848)
Supplement: Multimedia Appendix 2 [file aging_v6i1e39848_app2.docx]

Supplementary table 2: List of studies excluded during full text screening

| Title | Reason for exclusion |
| --- | --- |
| Association of Social Media Use With Social Well-Being, Positive Mental Health, and Self-Rated Health: Disentangling Routine Use From Emotional Connection to Use | Not evaluative |
| The relation between social network site usage and loneliness and mental health in community-dwelling older adults | Not evaluative |
| Health-related difficulty in internet use among older adults: Correlates and mediation of its association with quality of life through social support networks | Not evaluative |
| Patient Portal Utilization Among Ethnically Diverse Low Income Older Adults: Observational Study | Not evaluative |
| Older Adult Internet Use and eHealth Literacy | Not evaluative |
| Influence of previous work experience and education on Internet use of people in their 60s and 70s | Not evaluative |
| A home-based individualized information communication technology training program for older adults: a demonstration of effectiveness and value | No outcome on reducing loneliness or social isolation |
| Multi-stakeholder perspectives on information communication technology training for older adults: implications for teaching and learning | No outcome on reducing loneliness or social isolation |
| Comparing local policy practices to implement ICT-based home care services for aging-in-place in Finland, France, Italy, Spain, and Sweden | No outcome on reducing loneliness or social isolation |
| Understanding the use and non-use of social communication technologies by older adults: A qualitative test and extension of the UTAUT model | Not evaluative |
| Enhancing social engagement of older adults through technology | Not evaluative |
| Implementing reverse mentoring to address social isolation among older adults | No outcome on reducing loneliness or social isolation |
| Technology-Mediated Communication in Familial Relationships: Moderated-Mediation Models of Isolation and Loneliness | Not evaluative |
| Willingness and performance of older adults using Information ana Communication Technologies for cognitive activity and social interaction | No outcome on reducing loneliness or social isolation |
| Perceived Usefulness and Easiness of Information and Communication Technologies and Volunteering among Older Adults | Not evaluative |
| Let's play together: Effects of video-game play on intergenerational perceptions among youth and elderly participants | No outcome on reducing loneliness or social isolation |
| Falling off the bandwagon? Exploring the challenges to sustained digital engagement by older people | Not evaluative |
| Keeping silver surfers on the crest of a wave – older people's ICT learning and support needs | Not evaluative |
| Reducing the Digital Divide: Connecting Older Adults to iPad Technology | Not evaluative |
| Breaking Social Isolation Amidst COVID-19: A Viewpoint on Improving Access to Technology in Long-Term Care Facilities | Not evaluative |
| User Requirements for Technology to Assist Aging in Place: Qualitative Study of Older People and Their Informal Support Networks | Not evaluative |
| The iPad project: Introducing iPads into care homes in the UK to support digital inclusion | No outcome on reducing loneliness or social isolation |
| Information and communicative technology use enhances psychological well-being of older adults: the roles of age, social connectedness, and frailty status | Not evaluative |
| Design of a mobile social community platform for older chinese people in urban areas | No outcome on reducing loneliness or social isolation |
| SeniorTec: Senior Tutorial Program to Teach Technologies to Students | No outcome on reducing loneliness or social isolation |
| Combating isolation through technology in older people | Not evaluative |
| Social exclusion and information and communication technologies: Lessons from studies of single parents and the young elderly | Not evaluative |
| The dual impact of online communication on older adults' social connectivity | Not evaluative |
| Creating a conversational context through video blogging: A case study of Geriatric1927 | No outcome on reducing loneliness or social isolation |
| Videophone communication between residents and family: a case study | No outcome on reducing loneliness or social isolation |
| Internet use and well-being in older adults | Not evaluative |
| Exploring the perceptions of people with dementia about the social robot PARO in a hospital setting | Not two-way digital intergenerational program |
| The implications of information and communication technology use for the social well-being of older adults | Not evaluative |
| Technology Access and Use, and Their Associations With Social Engagement Among Older Adults: Do Women and Men Differ? | Not evaluative |
| Factors affecting information and communication technology use and eHealth literacy among older adults in the US and South Korea | Not evaluative |
| A Mobile Game for the Social and Cognitive Well-Being of Elderly People in China | No outcome on reducing loneliness or social isolation |
| Facilitating the adoption of digital technologies by the elderly | No outcome on reducing loneliness or social isolation |
| Being old in an always-on culture: Older people's perceptions and experiences of online communication | Not evaluative |
| Problematic Social Media Use and Perceived Social Isolation in Older Adults: A Cross-Sectional Study | Not evaluative |
| Televisits: sustaining long distance family relationships among institutionalized elders through technology | No outcome on reducing loneliness or social isolation |
| Acceptance of televideo technology by adults aging with a mobility impairment for health and wellness interventions | No outcome on reducing loneliness or social isolation |
| Using telepresence for social connection: views of older people with dementia, families, and health professionals from a mixed methods pilot study | No outcome on reducing loneliness or social isolation |
| Older adults' attitudes and barriers toward the use of mobile phones | Not evaluative |
| Intergenerational relationships and community computer training: Overcoming the digital divide | No outcome on reducing loneliness or social isolation |
| Engaging technology-resistant elderly people: Empirical evidence from an ICT-enabled social environment | No outcome on reducing loneliness or social isolation |
| Older Adults' Reasons for Using Technology while Aging in Place | Not evaluative |
| Understanding changes and stability in the long-term use of technologies by seniors who are aging in place: a dynamical framework | Not evaluative |
| Mobile applications in an aging society: Status and trends | Not evaluative |
| "Call a Teenager… That's What I Do!" - Grandchildren Help Older Adults Use New Technologies: Qualitative Study | Not evaluative |
| Mobile Support for Older Adults and Their Caregivers: Dyad Usability Study | No outcome on reducing loneliness or social isolation |
| Exploring the Relationship Between Online Social Network Site Usage and the Impact on Quality of Life for Older and Younger Users: An Interaction Analysis | Not evaluative |
| Tablet-Based Well-Being Check for the Elderly: Development and Evaluation of Usability and Acceptability | No outcome on reducing loneliness or social isolation |
| User-Centred Approach to Design an Online Social Support Platform for Seniors : Identification of Users' Types and Their Requirements | Not evaluative |
| Discontinued Information and Communication Technology Usage among Older Adults in Continuing Care Retirement Communities in the United States | No outcome on reducing loneliness or social isolation |
| Computer Training for Seniors: An Academic-Community Partnership | No outcome on reducing loneliness or social isolation |
| Maximizing Computer Use Among the Elderly in Rural Senior Centers | No outcome on reducing loneliness or social isolation |
| Reactions to a remote-controlled video-communication robot in seniors' homes: a pilot study of feasibility and acceptance | No outcome on reducing loneliness or social isolation |
| "Exploring the potential of virtual worlds in engaging older people and supporting healthy aging": Erratum | Not evaluative |
| Design for social media engagement: Insights from elderly care assistance | No outcome on reducing loneliness or social isolation |
| Internet use and loneliness in older adults | Not evaluative |
| Unequal access: Applying Bourdieu's practice theory to illuminate the challenges of ICT use among senior citizens in Singapore | Not evaluative |
| Seniors, entitlements and social media---oh my! | Not evaluative |
| Attitudes Toward and Predictors of Videoconferencing Use Among Frequent Family Visitors to Nursing Home Residents in Taiwan | Not evaluative |
| Getting Grandma Online: Are Tablets the Answer for Increasing Digital Inclusion for Older Adults in the U.S.? | Not evaluative |
| Social Support and “Playing Around” | Not evaluative |
| Perceptions of a Specific Family Communication Application among Grandparents and Grandchildren: An Extension of the Technology Acceptance Model | No outcome on reducing loneliness or social isolation |
| Older adult Internet super-users: Counsel from experience | Not evaluative |
| "Who over 65 is online?" Older adults' dispositions toward information communication technology | Not evaluative |
| Building social inclusion for rural, older people using ICTs | Not evaluative |
| The experiences of older adults who engage in social media: A generic qualitative study | Not evaluative |
| When Going Digital Becomes a Necessity: Ensuring Older Adults' Needs for Information, Services, and Social Inclusion During COVID-19 | Not evaluative |
| Older adults' use of smart phones: an investigation of the factors influencing the acceptance of new functions | Not evaluative |
| Advancing health information technology roadmaps in long term care | No outcome on reducing loneliness or social isolation |
| The effectiveness of a web 2.0 physical activity intervention in older adults - a randomised controlled trial | No outcome on reducing loneliness or social isolation |
| The Virtual Care Farm: A Preliminary Evaluation of an Innovative Approach to Addressing Loneliness and Building Community through Nature and Technology | No outcome on reducing loneliness or social isolation |
| Intergenerational telementoring for the promotion of social relationships | No outcome on reducing loneliness or social isolation |
| The impact of international videoconferencing among older adults and secondary students | No outcome on reducing loneliness or social isolation |
| Education in civic participation: Children, seniors and the challenges of an intergenerational information and communications technology program | No outcome on reducing loneliness or social isolation |
| A socialization intervention in remote health coaching for older adults in the home | No outcome on reducing loneliness or social isolation |
| Video-calls to reduce loneliness and social isolation within care environments for older people: an implementation study using collaborative action research | No outcome on reducing loneliness or social isolation |
| Using Telehealth Groups to Combat Loneliness in Older Adults Through COVID‐19 | No outcome on reducing loneliness or social isolation |
| The association between use of online social networks sites and perceived social isolation among individuals in the second half of life: results based on a nationally representative sample in Germany | Not evaluative |
| Social pathways to health: On the mediating role of the social network in the relation between socio-economic position and health | Not digital |
| Dual Use of a Patient Portal and Clinical Video Telehealth by Veterans with Mental Health Diagnoses: Retrospective, Cross-Sectional Analysis | Not two-way digital intergenerational program |
| Readiness to accept health information and communication technologies: A population-based survey of community-dwelling older adults | Not evaluative |
| Computers and caregiving: reaching out and redesigning interventions for homebound older adults and caregivers | Not evaluative |
| Online engagement and cognitive function among older adults | Not evaluative |
| The uses and gratifications of online care pages: a study of CaringBridge | Not in elderly |
| La participation sociale dans les résidences privées pour aînés | Not digital |
| Going Online to Stay Connected: Online Social Participation Buffers the Relationship Between Pain and Depression | Not evaluative |
| Smart Environments and Social Robots for Age-Friendly Integrated Care Services | Not evaluative |
| Potential Applications of Smart Multifunctional Wearable Materials to Gerontology | Not digital |
| Caregiver perspectives on a smart home-based socially assistive robot for individuals with Alzheimer's disease and related dementia | Not two-way digital intergenerational program |
| Involving service users and their carers as equal partners in a project using electronic communication | Not in elderly |
| Migration, Aging, and Digital Kinning: The Role of Distant Care Support Networks in Experiences of Aging Well | Not evaluative |
| Can videoconferencing affect older people's engagement and perception of their social support in long-term conditions management: a social network analysis from the Telehealth Literacy Project | Not two-way digital intergenerational program |
| Delivering patient education by group videoconferencing into the home: Lessons learnt from the Telehealth Literacy Project | Not two-way digital intergenerational program |
| 15 Smartphone Apps for Older Adults to Use While in Isolation During the COVID-19 Pandemic | Not two-way digital intergenerational program |
| Community-based research and approaches to loneliness prevention | Not digital |
| Although we're isolated, we're not really isolated': The value of information and communication technology for older people in rural Australia | Not evaluative |
| Internet use and mental health/well-being in old age: Exploring the roles of social integration and social support | Not evaluative |
| Programs to combat loneliness in the institutionalised elderly: A review of the scientific literature | Not English |
| Coaching Through Technology: A Systematic Review into Efficacy and Effectiveness for the Ageing Population | Not two-way digital intergenerational program |
| Impact of computer training courses on reduction of loneliness of older people in Finland and Slovenia | Not two-way digital intergenerational program |
| Tangible user interface for social interactions for the elderly: A review of literature | Not two-way digital intergenerational program |
| Uses and Gratifications of Computers in South African Elderly People | Not two-way digital intergenerational program |
| An intergenerational e-mail pal project on attitudes of college students toward older adults | No outcome on reducing loneliness or social isolation |
| Reactions to COVID-19, information and technology use, and social connectedness among older adults with pre-frailty and frailty | Not evaluative |
| Use of Gerontechnology to Assist Older Adults to Cope with the COVID-19 Pandemic | Not evaluative |
| Roles of Smartphone App Use in Improving Social Capital and Reducing Social Isolation | Not in elderly |
| A Web-based health promotion program for older workers: randomized controlled trial | Not two-way digital intergenerational program |
| Examining the Roles of Technology in Aging and Quality of Life | Not evaluative |
| Impact of internet use on loneliness and contact with others among older adults: cross-sectional analysis | Not evaluative |
| ICT4Life Integrated Care Platform | No full text |
| An investigation of the associations between social connectedness and Internet usage for older adults | Not evaluative |
| Coping with being cooped up: Social distancing during COVID-19 among 60+ in the United States | Not evaluative |
| Social media use and physical activity: Searching for opportunities to connect adolescents and older adults for health promotion | No full text |
| My Little Smart Personal Assistant: A Co-Designed Solution to Ensure an Optimized Ageing-Well at Home in Rural European Settings | Not evaluative |
| "Thanks to the Internet, we remain a family": ICT domestication by elderly immigrants and their families in Israel | Not evaluative |
| Connecting activities on Social Network Sites and life satisfaction: A comparison of older and younger users | No outcome on reducing loneliness or social isolation |
| Technology Access and Use, and Their Associations With Social Engagement Among Older Adults: Do Women and Men Differ? | Duplicate |
| Digital technology to enable aging in place | No outcome on reducing loneliness or social isolation |
| Instapals: Reducing ageism by facilitating intergenerational contact and providing aging education | No outcome on reducing loneliness or social isolation |
| The significance of digital citizenship in the well-being of older migrants | Not evaluative |
| We don't even have Wi-Fi': a descriptive study exploring current use and availability of communication technologies in residential aged care | Not evaluative |
| Effects of online social networking on the cognitive, social, and emotional health of older adults | Duplicate |
| Seniors' online communities: a quantitative content analysis | Not evaluative |
| The fun culture in seniors' online communities | Not evaluative |
| Aging Well in the Digital Age: Technology in Processes of Selective Optimization with Compensation | Not evaluative |
| The Dual Roles Technology Plays in Leisure: Insights from a Study of Grandmothers | Not evaluative |
| Togetherness in another way: Internet as a tool for togetherness in everyday occupations among older adults | Not evaluative |
| Interventions to address social connectedness and loneliness for older adults: a scoping review | Not digital |
| Interventions to address social connectedness and loneliness for older adults: a scoping review | Duplicate |
| Impacts of Urbanization and ICT Use on Loneliness Among the Elderly in Israel | Not evaluative |
| Barriers to the adoption of cell phones for older people with impairments in the USA: Results from an expert review and field study | Not evaluative |
| Mobile Phone, Computer, and Internet Use Among Older Homeless Adults: Results from the HOPE HOME Cohort Study | Not evaluative |
| Use of Information and Communication Technology (ICT) Devices Among the Oldest-Old: Loneliness, Anomie, and Autonomy | Not evaluative |
| Confidant Network and Quality of Life of Individuals Aged 50+: The Positive Role of Internet Use | Not evaluative |
| Information and Communication Technology Use Is Related to Higher Well-Being Among the Oldest-Old | Not evaluative |
| Facebook as a source of social connectedness in older adults | Not evaluative |
| Internet use as a predictor of sense of community in older people | Not evaluative |
| Decreasing loneliness and social disconnectedness among community-dwelling older adults: The potential of information and communication technologies and ride-hailing services | Not evaluative |
| Does social network site use buffer against well-being loss when older adults face reduced functional ability? | Not evaluative |
| Building social inclusion for rural, older people using ICTs | Duplicate |
| How to market use of social media to improve older adults' health | Not evaluative |
| The lived experience of social support for older adults in a computer-mediated environment: A phenomenological research study | Not evaluative |
| Facebook use and its role in shaping access to social benefits among older adults | Not evaluative |
| Assessing the impact of Social Networking Site use on older people's loneliness and social isolation. A randomized controlled trial: The Aging in a Networked Society-Social Experiment Study (ANS-SE) | Not evaluative |
| Can Digital Technology Enhance Social Connectedness Among Older Adults? A Feasibility Study | Duplicate |
| Intergenerational digital engagement: A way to prevent social isolation during the COVID-19 crisis | Not evaluative |
| Effects of a smartphone-based videoconferencing program for older nursing home residents on depression, loneliness, and quality of life: a quasi-experimental study | Duplicate |
| Practical issues related to the implication of elderlies in the design process-The case of a Living Lab approach for designing and evaluating social TV services | No outcome on reducing loneliness or social isolation |
| TV as an experience conveyer for better acceptance of ICT services by older adults | No outcome on reducing loneliness or social isolation |
| Everyday use of computer-mediated communication tools and its evolution over time: An ethnographical study with older people | Not evaluative |
| Determinants of user acceptance of a specific social platform for older adults: An empirical examination of user interface characteristics and behavioral intention | No outcome on reducing loneliness or social isolation |
| Impact of a Virtual Learning Program on Social Isolation for Older Adults | No outcome on reducing loneliness or social isolation |
| A Cognitive Social Media Training Program and Intergenerational Learning: A Pilot Study With Older Adults and Speech- Language Pathology Graduate Students | No outcome on reducing loneliness or social isolation |
| To Boldly Go Online: Empowering Elders to Connect Socially with Technology | No outcome on reducing loneliness or social isolation |
| Social support is associated with technology use in old age | Not evaluative |
| Evaluation of an Assistive Telepresence Robot for Elderly Healthcare | Not two-way digital intergenerational program |
| A systematic review of gamification techniques applied to elderly care | Not evaluative |
| From dinner table to digital tablet: Technology's potential for reducing loneliness in older adults | No full text |
| Potential of telepresence robots to enhance social connectedness in older adults with dementia: an integrative review of feasibility | Not two-way digital intergenerational program |
| Internet Programming to Reduce Loneliness and Social Isolation in Aging | Not two-way digital intergenerational program |
| Social prescribing: combating loneliness is everyone's business | Not two-way digital intergenerational program |
| Exploring the Use of Technology for Active Aging and Thriving | Not two-way digital intergenerational program |
| Grandparents use of new communication technologies in a European perspective | Not evaluative |
| When your world gets smaller: how older people try to meet their social needs, including the role of social technology | Not two-way digital intergenerational program |
| Older Adults Perceptions of Technology and Barriers to Interacting with Tablet Computers: A Focus Group Study | Not evaluative |
| Older Adults Experiences of Learning to Use Tablet Computers: A Mixed Methods Study | No outcome on reducing loneliness or social isolation |
| Silver surfers from a European perspective: technology communication usage among European seniors | Not two-way digital intergenerational program |
| Internet Use and Loneliness of Older Adults Over Time: the mediating effect of social contact | Not evaluative |
| The relationships that matter: Social network site use and social wellbeing among older adults in the United States of America | Not two-way digital intergenerational program |
| Attitudes Towards and Use of Information and Communication Technologies (ICTs) Among Older Adults in Italy and Sweden: the Influence of Cultural Context, Socio-Demographic Factors, and Time Perspective | Not two-way digital intergenerational program |
| Evaluation of ICT supported integrated healthcare for frail patients with comorbidities: Baseline assessment of the CareWell Project | No full text |
| Stay Tuned: The Role of ICTs in Elderly Life | No outcome on reducing loneliness or social isolation |
| New app being developed to help reduce loneliness in elderly | No full text |
| "thanks to Facebook, getting old isn't that bad and i am not all alone in this world": An investigation of the effect of Facebook use on mattering and loneliness among elder orphans | Not evaluative |
| Dumb 'Skype' on wheels in care homes-a new design | No outcome on reducing loneliness or social isolation |
| Social media: an isolated older person's friend--or foe? | No full text |
| Isolation and gerontechnology: Computer-assisted social engagement | No full text |
| Exploring the relationship between technology usage and social inclusion for older adults with intellectual disability in Ireland | No full text |
| An Evaluation of a Low-Intensity Cognitive Behavioral Therapy mHealth-Supported Intervention to Reduce Loneliness in Older People | Not two-way digital intergenerational program |
| "This phone saved my life": Older persons' experiences and appraisals of an mHealth intervention aimed at addressing loneliness | Not two-way digital intergenerational program |
| Pilot testing the effectiveness of the healthy ageing supported by internet and community programme for promoting healthy lifestyles for people over 65 years of age | Not two-way digital intergenerational program |
| Internet Programming to Reduce Loneliness and Social Isolation in Aging | Not two-way digital intergenerational program |
| An innovative plan to combat the downside of social isolation | Not two-way digital intergenerational program |
| The Cafe-multimedia: A promising psychosocial intervention for frail older adults | No full text |
| Exploring the impact of a group-based tablet-PC training program in older adults | Not English |
| Ah, Look at All the Lonely People (And Resultant Health Problems Thereof) | No full text |
| Technology-based health and wellness self-management for older adults | No full text |
| Reducing health disparities in older people through assistive technology | No full text |
| ESeniors: An intergenerational model to enhance computer skills for older adults | No full text |
| Social media a new medicine? | Not two-way digital intergenerational program |
| Aging in computer-mediated community: The importance of online connections for older adults | No full text |
| Reconnect interface for older adults: A motion-based interface for older adults to communicate with their family | Not two-way digital intergenerational program |
| Using video-calls to combat loneliness in old age | No full text |
| Implementation of CareTV in care for the elderly: the effects on feelings of loneliness and safety and future challenges. | Duplicate |
| Facebook exposure as a tool for reducing negative emotions among older adults | No outcome on reducing loneliness or social isolation |
