# Supplementary material for: Digital Intergenerational Program to Reduce Loneliness and Social Isolation Among Older Adults: Realist Review
Source: JMIR Aging. 2023 Jan 4;6:e39848. doi: 10.2196/39848 (PMC9850285; doi:10.2196/39848)
Supplement: Multimedia Appendix 3 [file aging_v6i1e39848_app3.docx]

Supplementary table 3: MMAT appraisal for documents evaluating digital intergenerational program

| **Program** | **Document title** | **Type of study** | **Responses for each MMAT question** | | | | |
| --- | --- | --- | --- | --- | --- | --- | --- |
|  |  |  | **1** | **2** | **3** | **4** | **5** |
| ACTION | A pilot study of how information and communication technology may contribute to health promotion among elderly spousal carers in Norway [62] | Mixed methods | Yes | Yes | Yes | Yes | Yes |
| ACTION (redesigned) | An Internet-based videoconferencing system for supporting frail elderly people and their carers [63] | Mixed methods | No | Yes | Yes | Can't tell | No |
| ACTIVE | Introducing Technology for Thriving in Residential Long-Term Care [53] | Qualitative | Yes | Yes | Yes | Yes | Yes |
| AGES 2.0 | Activating and guiding the engagement of seniors with online social networking: Experimental findings from the AGES 2.0 project [49] | Quantitative RCT | Yes | Yes | Yes | Can't tell | No |
| AO | The supportive network: rural disadvantaged older people and ICT [74] | Qualitative | Yes | Yes | Yes | Yes | Yes |
| Collage and Storytelling | Supporting young children's communication with adult relatives across time zones [72] | Qualitative | Can't tell | Yes | Yes | Yes | Yes |
| Demiris et al | Use of videophones for distant caregiving: an enriching experience for families and residents in long-term care [59] | Qualitative | Can't tell | Yes | Yes | Yes | Yes |
| Digital Age | DIGITAL AGE PROJECT EVALUATION REPORT [60] | Mixed methods | Yes | Yes | Yes | Yes | Yes |
| Digital Age | Linking Generations Northern Ireland Evaluation of the Digital Age Report [81] | Mixed methods | Yes | Yes | Yes | Yes | Yes |
| [Esc@pe](mailto:Esc@pe) | Escape loneliness by going digital: a quantitative and qualitative evaluation of a Dutch experiment in using ECT to overcome loneliness among older adults [76] | Mixed methods | Yes | Yes | Yes | Yes | Yes |
| InTouch | Communication technology adoption among older adult veterans: the interplay of social and cognitive factors [64] | Mixed Methods | Yes | Yes | Yes | Yes | Yes |
| LINE | Effects of a smartphone-based videoconferencing program for older nursing home residents on depression, loneliness, and quality of life: a quasi-experimental study [79] | Quantitative non-randomized | Yes | Yes | Yes | Yes | Yes |
| Loi et al | Can a short internet training program improve social isolation and self-esteem in older adults with psychiatric conditions? [58] | Quantitative non-randomized | Can't tell | Yes | Yes | Yes | Yes |
| Media parcels | Promoting social connection and deepening relations among older adults: Design and qualitative evaluation of media parcels [65] | Qualitative | Yes | Yes | Yes | Yes | Yes |
| MSN or Skype | Videoconference program enhances social support, loneliness, and depressive status of elderly nursing home residents [80] | Quantitative non-randomized | Yes | Yes | Yes | Yes | Yes |
| MSN or Skype | Changes in depressive symptoms, social support, and loneliness over 1 year after a minimum 3-month videoconference program for older nursing home resident [82] | Quantitative non-randomized | Yes | Yes | Yes | Yes | Yes |
| Neves et al | Can digital technology enhance social connectedness among older adults? A feasibility study [57] | Qualitative | Yes | Yes | Yes | Yes | Yes |
| Plymouth SeniorNet | Older people going online: its value and before-after evaluation of volunteer support [66] | Quantitative non-randomized | Yes | Yes | Yes | Yes | Yes |
| PRISM | Improving Social Support for Older Adults Through Technology: Findings From the PRISM Randomized Controlled Trial [67] | Quantitative RCT | No | Yes | Yes | Yes | Yes |
| Skype | Videoconferencing intervention for depressive symptoms and loneliness in nursing home elders [54] | Quantitative non-randomized | Yes | Yes | Yes | Yes | Yes |
| Skype on Wheel | Skype on Wheels: Implementation of video-calls to reduce feelings of loneliness and social isolation for older people living in care [61] | Qualitative | Yes | Yes | Yes | Yes | Yes |
| Skype on Wheel | Video-calls to reduce loneliness and social isolation within care environments for older people: an implementation study using collaborative action research [83] | Qualitative | Yes | Yes | Yes | Yes | Yes |
| StoryBox | Supporting Communication between Grandparents and Grandchildren through Tangible Storytelling Systems [71] | Mixed methods | No | Yes | Yes | Can't tell | No |
| Tech Allies | In-home technology training to reduce social isolation among older adults: Findings from the tech allies program [78] | Quantitative RCT | Can't tell | Yes | Yes | Can't tell | Yes |
| Tele-BA | Improving Social Connectedness for Homebound Older Adults: Randomized Controlled Trial of Tele-Delivered Behavioral Activation Versus Tele-Delivered Friendly Visits [75] | Quantitative RCT | Can't tell | Yes | Yes | Can't tell | Yes |
| Tele-BA | Effect of Telehealth Treatment by Lay Counselors vs by Clinicians on Depressive Symptoms Among Older Adults Who Are Homebound: A Randomized Clinical Trial [84] | Quantitative RCT | Can't tell | Yes | Yes | Can't tell | Yes |
| Telesenior | Effectiveness of video-telephone nursing care for the homebound elderly [68] | Quantitative non-randomized | Yes | Yes | Yes | Can't tell | Yes |
| Tlatoque | Enriching in-person encounters through social media: A study on family connectedness for the elderly [69] | Qualitative | Yes | Yes | Yes | Yes | Yes |
| White et al | A randomized controlled trial of the psychosocial impact of providing internet training and access to older adults [77] | Mixed methods | Yes | Yes | Yes | Yes | Yes |
| Williams et al | Using computer-mediated communication to reduce loneliness in older adults [73] | Quantitative non-randomized | Yes | Yes | Yes | Yes | Yes |
| You, me & TV | "You, me & TV"-Fighting social isolation of older adults with Facebook, TV and multimodality [70] | Mixed methods | No | No | Yes | Can't tell | No |

Abbreviation

MMAT: Mixed Methods Appraisal Tool; RCT: Randomized controlled trials
