# Supplementary material for: Digital Intergenerational Program to Reduce Loneliness and Social Isolation Among Older Adults: Realist Review
Source: JMIR Aging. 2023 Jan 4;6:e39848. doi: 10.2196/39848 (PMC9850285; doi:10.2196/39848)
Supplement: Multimedia Appendix 4 [file aging_v6i1e39848_app4.docx]

Supplementary table 4: Candidate S-C-M-O configurations for digital intergenerational program based on the authors’ description

| **Program** | **Strategy** | **Context** | **Mechanism** | **Main outcome** |
| --- | --- | --- | --- | --- |
| ACTION [62] | Participants received a modern broadband-linked personal computer, and an information and communication technology course consisting of three 3-hour classes dispersed over a 3-week period. | 60 years of age or older, had been caring for elderly person for less than 2 years, were a computer novice and had Norwegian as their first language | Gain support | There was a positive and significant change in scores with regards to contact with family and friends (P= 0.036) and a sense of social support from other persons (P= 0.010). |
| ACTION (redesigned) [63] | Participants could access a variety of multimedia information programs in the ACTION database and use the videoconferencing device for consultation and social purposes. Internet access was also available and could be used to view information resources and other services. Internet and email facilities also increased the opportunities for social networking. | Older than 66 years old | The video calls with nurses are more social in nature. | Seven of the participants (88%) reported that the system very much reduced their sense of loneliness and isolation. |
| ACTIVE [53] | Provided an Internet connected tablet free of charge, to use for an unlimited period of time. The iPad was set up with an individual user account, including e-mail, Apple-ID, Skype-ID, passwords, and codes. This information was given in writing to each participant. To avoid overwhelming the participants, a carefully selected, smaller set of basic apps was installed | Older adults with little prior knowledge of tablets, living in a supported, senior apartment or residential care facility. | An important dimension of the intervention was the scheduled, regular opportunities for follow-up that provided predictability, stability, and regularity to when support was available. | The nurses observed that being able to use an iPad and the Internet facilitated thriving and social participation, regardless of level of performance. |
| AGES 2.0 [49] | The participants received a customized computer platform with a simplified touch-screen interface ("EasyPC") and any necessary broadband infrastructure. Care technologists administered training. | Clients of a large not-for-profit care organization | Feedback from carers suggested that some individuals do not find this a positive and enhancing experience, and preferred not to engage with the computer they were given. The study also found that feelings of self-competence, social engagement, and maintenance of identity were critical to intervention's success. | No effect of training on subjective feelings of loneliness. |
| AO[74] | Provided Apple iPad with cellular access, along with vouchers for data access throughout the project, and $30 App Store card for buying applications. Approximately one month after the participants had received the tablets, there was more intensive one-to-one training on the device combined with goal setting, to establish what services and communication methods most appealed to each participant. | Clients of the Assertive Outreach program (provides long term case management support for people living independently in the community with histories of homelessness, social isolation and multiple and complex needs), be over the age of 50 and self-identify as being able to read and write English to a competent standard | Most participants were very socially isolated in real life, and often had very fractured family histories. Technological barriers include (1) Tablet system wide notifications, user interface conventions that were not intuitive and user interface elements that led to participants disabling the tablet, and (2)  Issues with connecting to cellular network and maintaining pre-paid data plans. | Establishing even a small online social network proved very difficult in many cases. However, there were a small number of exceptions. |
| Collage and Storytelling [72] | The system used combines the “Collage”  component and the “Storytelling”  component. | Grandparents | Collage mediates play in both synchronous and asynchronous settings while Storytelling mediates oral storytelling in a synchronous setting only. | The system successfully facilitated contact between the grandparents and grandchildren as both families enjoyed using the system and felt they were closer to their remote family members after having used the system. |
| Demiris et al [59] | The videophone can display three kinds of real-time images during a videocall: self, other party, and a combination of both, depending on user preference. It plugs into a regular telephone and does not interfere with its use. A videocall is possible only when both parties have videophone units and consent to a videocall (by pressing the video button). | Older than age 65 | Videophone promotes a social presence for the resident and family member. | The videophone communication contributed positively in reducing feelings of isolation and loneliness for residents. |
| Digital Age [60] | Digital Age consisted of a free, in-house, 10-week IT course for residents. The project also provided free IT hardware for each participating housing scheme, free online digital toolkits, and a series of intergenerational digital projects to encourage links between older and younger people, further develop residents’ digital capabilities and help to sustain the programme beyond the project lifetime. | Older people living in social housing | Increase channels of communication; improve access to information, services, hobbies and interests; and reduce social isolation through interaction between different generations. | The key benefit to the participants is their increased ability to keep in touch with friends and family using technology. |
| [Esc@pe](mailto:Esc@pe) [76] | At the start of the project, the participants were given five two-hour lessons at home by experienced teachers, all volunteers of SeniorWeb Eindhoven. During these lessons the participants learned how to e-mail and how to use the Internet. During the rest of the project, the participants were supported and coached by visiting volunteers from the Red Cross or De Zonnebloem who had also paid home visits to the participants once every two or three weeks prior to the start of the pilot project. | Volunteer home visitors of the Red Cross and De Zonnebloem | The use of PC and Internet reduce feelings of loneliness by improving the participants' social lives and distracting them from their loneliness experience. | The participants in intervention group have significantly reduced total score loneliness and emotional loneliness, but not the control group. |
| InTouch [64] | Participants and volunteers were each given an iPad with the InTouch app on it, as well as a detailed instructions manual, for the 12-week study period. In addition to the support of their volunteer, participants were given a direct link through the app to a staff member (Activities Manager), who could assist between the volunteer visits. Participants were paired 1:1 with volunteers who taught them how to use InTouch, meeting weekly for 12 weeks (in addition to user manual and peer support). | Senior participants from Veterans’ Centre | Physical health conditions, previous experience with technology (with tech-savvy participants finding little use for InTouch), and social motivators appeared to influence how participants adopted/ experienced InTouch, as well as the effect it had on their social lives and overall well-being. | Most participants described increased frequency of communication and/or strength of relationships as a result of using InTouch. |
| LINE [79] | The participants interacted with their family members once a week for 6 months using a smartphone and "LINE" application. We provided some discussion topics to nurses and the participants, such as their meals, organised activities and ‘news’ on nursing home life. | Residents were recruited if they: 1) were over 60 years of age; 2) had a Mini-Mental State Examination (MMSE) score ≥ 16 for residents with no formal education or MMSE> 20 for residents with at least a primary school education; 3) had no experience in using smartphones for videoconferencing with people prior to this research; and 4) both residents and their family members agreed to participate in the study. | Smartphones may optimize the interactions between nursing home residents and their families. | Controlling for the effects of age and frequency of in-person family visits, the changes in scores for loneliness were significantly lower for the participants in the intervention group compared to the control group (all p-values < .001), demonstrating that feelings of loneliness decreased significantly at 1 month, 3 months and 6 months. |
| Loi et al [58] | Structured six week, twice weekly program of 45 minutes duration based on a local program used for older adults (Internet for Seniors). Apple iPads were used. | Older adults with psychiatric conditions | Touchscreen technology such as iPads may be easier to use as it has a relatively large screen which has the ability to increase the font size and icons for improved visibility, and by using an onscreen keyboard is less reliant on dexterity. | No statistically significant difference in social isolation post intervention |
| Media parcels [65] | First, a facilitator, upon specific requests to participants, collects media and wraps them in text commentary, bringing out their memories and meaning. Next, the facilitator passes the wrapped media parcel to a target person, who in turn unwraps them. | (1) aged 60 years or older; (2) lives alone; (3) is able to nominate at least two other social contacts who are able to participate. | The media parcels facilitate reflection and communication between parties and triggering further conversation outside the system. The human facilitator in the loop, with a background in clinical psychology, authored requests and selected responses to maximise positive effects on relationships. | All participants reported feeling closer to each other and contacting each other more than usual during the Media Parcels trial. |
| MSN or Skype [80] | The videoconference program was designed for once a week (the in-person visiting frequency for the majority of families) and to last for three months to provide time for adjustment to a new program. The residents were helped to use the videoconference technology by a trained research assistant, who spent at least five min per week with the residents at the appointment time. The contact family member was the resident’s spouse, child, or grandchild. The software at the facilities was either MSN or Skype via a 2 M/256K wireless modem using a large (15.6 cm) laptop. | Residents of these nursing homes were recruited if they met the following criteria: (1) over 60 years old; (2) Mini-Mental State Examination (MMSE) score≥ 16 for participants with no formal education or MMSE>20 for elders with at least a primary school education; and (3) their residence floor had wireless Internet access. | Videoconferencing significantly reduced residents’ loneliness may be due to the language interaction with their family members. | Participants in the intervention group had lower mean loneliness scores at one week and three months after baseline than those in control group. |
| Neves et al [57] | An accessible iPad-based communication app that supports participants’ asynchronous communication with family and friends. The app allowed users to send and receive photos, audio, video, and text messages (sent messages were predefined to increase simplicity), whereas their contacts could respond using their own emails and devices. The interface offered large non-textual touch icons (no typing, only swiping/ tapping) to accommodate users with visual and motor impairments. Predeployment included an individual training session. | Residents at a retirement home | Active involvement of relatives or friends was crucial for adoption of the app, learning dynamics, and type and continuity of use. In addition, the app’s perceived usefulness and functionality seemed related to levels of acceptability and efficacy. Having geographically distant relatives can also amplify the app’s feasibility to enhance social connectedness. | Although the app increased sense of social interaction (communication frequency and type) with family and friends for 10 participants, only four reported high perceived social connectedness at post deployment. For the other 6 participants, the app did not make those relationships more meaningful as they only used the app for brief contact or follow-ups. Three participants indicated no changes in social interaction or connectedness. |
| Plymouth SeniorNet [66] | There were two main interventions—volunteers supported participants (1) one-on-one in their own homes, spending an average of 12 hours together over eight visits, and (2) in 90 small groups spending 12 hours with participants per group. Participants thought to be more physically isolated were allocated to one-on-one support. Sessions in both settings covered basic computer use, how to get online and search the Internet, online shopping, email, Skype or FaceTime, and online news and entertainment. Volunteers supported some participants in choosing and setting up equipment and broadband. The elapsed time for the intervention depended on an agreement between volunteers and participants about whether they had had sufficient support. | People aged 65 years and older | Conducting training and support sessions with participants may improve self-reported measures of Internet confidence, knowledge, and self-efficacy, which can ultimately lead to increased Internet use. Such tuition may be more effective if received from peers of similar age. | No significant difference in loneliness for one-to-one sessions. Reduced loneliness observed for those in group sessions. |
| PRISM [67] | Participants received a Lenovo “Mini Desktop” PC with a keyboard, mouse (or trackball for those who were unable to control a mouse), a 19″ LCD monitor, the PRISM software application, and a printer. Computers were linked to a secure server at the host site and free Internet access was provided through a wireless card. PRISM included: Internet access (with vetted links to sites such as NIHSeniorHealth.Gov), an annotated resource guide, a dynamic classroom feature, a calendar, a photo feature, E-mail, games, and online help. PRISM was built using an iterative user-centered design approach. All participants received 3 additional home visits for training. | Individuals aged 65 or older living alone in independent housing, who spoke English, had at least 20/60 vision with or without correction, and could read at the United States 6th grade level. They had minimal computer/ Internet use, were not employed or volunteering more than 5 hr/week, or spending more than 10 hr/week at a senior center or formal organization. | PRISM provided participants with access to a broad array of features beyond the Internet such as E-mail, games, and a dynamic classroom feature, which provided additional opportunities for social interaction and engagement. The content of PRISM features was chosen in terms of potential relevance to older adults, the features were easy to access (only requiring one “click” on the feature name on the homepage sidebar menu), and it was easy to shift among features. The system was also dynamic in the sense that participants had easy access to other information (e.g., through vetted links and videos) and the classroom was updated monthly. The system also provided opportunities to make new acquaintances with individuals who had similar interests. | At 6 months, in comparison to Binder participants, PRISM participants had a significantly greater decline in loneliness and greater increase in perceived social support. There was also a trend indicating that they experienced a greater decline in social isolation at 6 months. |
| Skype [54] | The Skype videoconferencing took place on a weekly basis for a total of 10 sessions over a 14-week period in a private room at the nursing home. | Older than age 65 | Videoconference can assist participants with the verbal and nonverbal elements of communication all while providing the participants with a means of developing social presence | The participants in intervention group felt a significantly decreased level of loneliness after videoconferencing with family members than before. |
| Skype on Wheel [61] | Students from local school and older adults across three care homes in engaged in Skype video-calls over a six-week study. Residents were supported by care staff; students accessed Skype from school laptops. A conversational aid was trialled with students to assist their conversations with an older generation. | Residents in care home | Not all family members can commit to video-call communication with their older relatives as seen in cycle one. Also, younger generations (grandchildren) may not be sure of how to communicate with their elderly relatives (especially those with dementia) resulting in poor sustainability of social interactions due to awkward or uncomfortable conversations | Care staff felt that the intervention using video-calls were useful in “building friendships” between older people and a younger generation, and so bridging the generational gap. Residents had the opportunity to talk about themselves and impart some knowledge and advice as ‘friends’ would normally do. Socialisation over time improved due to a useful conversational aid (prompt sheet) that provided topics for discussion and led to a more “comfortable experience” avoiding “pauses and silences in between”. |
| StoryBox [71] | The StoryBox allows sending of (1) pictures, (2) audio messages to  family members, and (3) feedback to experimenters. | Grandparents | StoryBox alleviates the barriers of communication between different generations. For young grandchildren, this often means the sharing of crafts, drawings, stickers and short exclamations. For grandparents, the  device provides a way to digitize analog memories, and use  handwriting for communication. | One family mentioned that the communication via StoryBox increased social connectedness between the grandparents and grandson. |
| Tech Allies [78] | Participants took part in eight weekly, 1:1 digital training sessions. Participants each received a tablet, a tablet case, a stylus, broadband access through a low-cost internet program for low-income households or a hot spot device, and a certificate of completion at the end of the program. A learner booklet was created for each participant, outlining curriculum topics by week, including step-by-step visual guides and practice exercises. | Isolated and lonely older adults over 65 years old | Participants were already facing many contextual factors in their daily lives, such as physical disability and a lack of close friends and living relatives, that made their loneliness more systemic and harder to change | The intervention group showed no change in loneliness, and marginally significant improvement in social support. |
| Tele-BA [75] | Tele-BA treatment delivered by lay counselors. Included: Tele-delivery equipment (a secure laptop with a Health Insurance Portability and Accountability Act–compliant videoconferencing platform and a 4G wireless card) plus instructions and all written session materials (for psychoeducation, handouts, and worksheets). | Recipients of home-delivered meals programs who reported feeling lonely | Tele-BA participants learn how to overcome barriers to social connectedness and to use skills for maintaining social connectedness over time, thus effects can sustain beyond the sessions. | Tele-BA participants reported significantly lower levels of loneliness as compared to videoconferenced friendly visit. |
| Telesenior[68] | The tele-nurses delivered psychosocial support and educational interventions based on three principles: contact and communication, safety and protection, and care mediation. Participants were supplied with an integrated home terminal, consisting of an alarm station, telephone, television, camera, and necklace transmitter in case of emergency. | 60 years of age or older, had a variety of chronic illnesses, had some degree of functional impairment, required assistance with activities of daily living, and lived in a geographical area with a well-developed two-way cable television network | The mechanism behind this improvement may be that the video-telephone intervention provided participants with a network of relationships in which the participants felt accepted, had common interests and concerns, and found help, advice, and support. The participants asked to be connected, through video-telephone, with their children who lived far away and with other participants in the telecare program. They used their television as a focal point for their daily routines. The television served as a “window to the outside world” and may have been a substitute for primary interpersonal communication and relationships, offering companionship, information, and entertainment, and thus possibly increasing their life satisfaction. | Men under 70 years of age who had low levels of social activity, moderate physical limitations, or severe physical pain showed significant changes on the measure change in emotional loneliness. For the measure change in social loneliness significant improvement was found for a subgroup who frequently needed nursing care and home help and had strong feelings of emotional loneliness and for a subgroup who had limited social functioning. Participants with extreme feelings of social and emotional loneliness and with a limited friends and family network showed positive significant improvement on the measure change in friends network. |
| Tlatoque[69] | Tlatoque communicates to Facebook site to expose photographs in the user's home and provides means of reciprocating information into Facebook. During the first two weeks of study, the participants received two training sessions. The first one was about the general features of Tlatoque, and the second one focused on the use of its feedback services. | Members of two extended families | Tlatoque enable relatives to integrate the participants into the social networking site, catalyzed in-person encounters, and enriched social interactions between the participants and their relatives. Moving outside the desktop the social information shared on the SNS and making it available in Tlatoque's not only helped enrich the offline social experience but also eased the adoption of the technology. | Overall, the frequency of contact with participants increased. |
| White et al [77] | Participants received nine hours of small group training in six sessions over two weeks. Computers were available for continued use over five months and the trainer was available two hours/week for questions. | Volunteers from four congregate housing sites and two nursing facilities | Relatively isolated and disabled older adults can reconnect, strengthen and broaden their connection with the outside world by incorporating computer technology into their lives. | There is no difference in change scores for loneliness scales between the intervention and control groups. |
| Williams et al [73] | The course on computer-mediated communication lasted for six 2- hour classes. Each course followed the same module: Introduction to computer mediated communication, email and instant messaging, microblogging: Twitter, social networking sites: Facebook, video chat, and online safety. | Aged 60 and above and qualify as lonely utilizing a loneliness scale. | The course increases the computer-mediated communication self-efficacy, leading to increased computer communication use. The short duration of the intervention may have limited the effectiveness of the intervention. | No significant difference in loneliness between before and after participating in the program. |
| You, me & TV [70] | The system has 3 main features (1) User feed; (2) Managing groups of friends; and (3) Photo viewing and sharing. Each task was performed several times by the user until the user agreed he/she understood and could perform them without help. Contact channels with the trial moderator were also established so that any doubt could be clarified by phone or with a technical visit. | Older adults | Photos from participants' past encourage them to re-establish contact with relatives, and current photographs of family allow participants to know more about their relatives' status and activities, especially relatives who live far away.  Kinect camera which captures printed photograph and send it as a digital memoir to their relatives on the network was seen, not only as an easier way to upload a photo than the traditional one, but also allowed both participants and relatives to revive old memories and favored reciprocity.  Awareness of what TV shows they are watching created new streams of conversations, which consequently lead to an increase in the number of interactions with their relatives. | Two out of three participants have increase in number of sharing actions – liking posts, posting, commenting, sharing TV content, sharing printed photos. |
